# Relationship Between Basic Properties of BOLD Fluctuations and Calculated Metrics of Complexity in the Human Connectome Project

**DOI:** 10.3389/fnins.2020.550923

**Published:** 2020-09-15

**Authors:** Shella Keilholz, Eric Maltbie, Xiaodi Zhang, Behnaz Yousefi, Wen-Ju Pan, Nan Xu, Maysam Nezafati, Theodore J. LaGrow, Ying Guo

**Affiliations:** ^1^Department of Biomedical Engineering, Georgia Institute of Technology, Emory University, Atlanta, GA, United States; ^2^Department of Biostatistics and Bioinformatics, Emory University, Atlanta, GA, United States

**Keywords:** resting state – fMRI, complexity systems, BOLD signal, frequency, entropy

## Abstract

Resting state functional MRI (rs-fMRI) creates a rich four-dimensional data set that can be analyzed in a variety of ways. As more researchers come to view the brain as a complex dynamical system, tools are increasingly being drawn from other fields to characterize the complexity of the brain’s activity. However, given that the signal measured with rs-fMRI arises from the hemodynamic response to neural activity, the extent to which complexity metrics reflect neural complexity as compared to signal properties related to image quality remains unknown. To provide some insight into this question, correlation dimension, approximate entropy and Lyapunov exponent were calculated for different rs-fMRI scans from the same subject to examine their reliability. The metrics of complexity were then compared to several properties of the rs-fMRI signal from each brain area to determine if basic signal features could explain differences in the complexity metrics. Differences in complexity across brain areas were highly reliable and were closely linked to differences in the frequency profiles of the rs-fMRI signal. The spatial distributions of the complexity and frequency metrics suggest that they are both influenced by location-dependent signal properties that can obscure changes related to neural activity.

## Introduction

Resting state functional magnetic resonance imaging (rs-fMRI; Biswal et al., 1995) is a popular tool for characterizing the functional architecture of the brain. Based on the relationships between the blood oxygenation level dependent (BOLD) signal from different areas, it is possible to identify functional networks and test for changes that distinguish between patient groups or that relate to cognition (Fox et al., 2005; Rombouts et al., 2005; Sorg et al., 2007; Smith et al., 2009; Yeo et al., 2011; Barch et al., 2015; Magnuson et al., 2015).

While rs-fMRI analysis has usually used correlation or similar metrics to calculate network-based properties like functional connectivity, it is also possible to look at voxel-level or parcel-level properties. The most prominent examples of this type of analysis are ALFF (Zang et al., 2007) and fALFF (Zou et al., 2008), which describe the power of the BOLD signal that lies in the low frequency range typically used for rs-fMRI (0.01–0.08 Hz). Areas with higher power and thus stronger low frequency fluctuations are thought to have higher spontaneous neural activity. In line with this hypothesis, areas like the posterior cingulate cortex (PCC) that exhibit high resting state metabolism in PET scans also have higher ALFF and fALFF than other cortical areas (Zou et al., 2008). Furthermore, these measures of low frequency power can discriminate between patient groups and healthy controls or between rest and task performance (Zang et al., 2007; Cui et al., 2019; Li et al., 2019; Zhou et al., 2019).

As interest in the brain as a complex system grows, researchers are increasingly applying more sophisticated analytical techniques to the rs-fMRI signal. Time-varying analysis (rather than measures averaged over the whole scan, like typical correlation-based functional connectivity) attempts to characterize the underlying low-dimensional structure of the brain’s dynamics (Hutchison et al., 2013; Keilholz et al., 2017; Preti et al., 2017). At the voxel/parcel level, concepts from non-linear dynamics like entropy, Hurst exponent and Lyapunov exponent have been used to characterize the complexity of the signal from each area, based on the hypothesis that the complexity of the BOLD signal carries information about the complexity of the underlying neural activity (Wang et al., 2011; Yang et al., 2013; Ciuciu et al., 2014; Jia et al., 2017; Wang Y. et al., 2018; Liu et al., 2018, 2019; Song et al., 2019; Nezafati et al., 2020). However, these metrics are difficult to interpret in the context of rs-fMRI. The relationship between the BOLD signal and neural activity relies on neurovascular coupling, which can vary across areas. Moreover, the BOLD signal itself is low in amplitude and easily contaminated with physiological noise or motion. In the absence of “ground truth” measurements of neural complexity, it is difficult to be confident that BOLD-based metrics of complexity are meaningful.

We approached this problem by asking whether metrics of complexity provide unique information beyond the most basic features of the BOLD signal: its mean, standard deviation, temporal signal to noise ratio (tSNR) and power spectrum. In the large dataset available from the Human Connectome Project (HCP), we look at the relationship between these fundamental features and the higher level metrics of correlation dimension, approximate entropy, and Lyapunov exponent. Finally, we examine variability in fundamental properties and complexity metrics across parcels and across individuals, and their reliability across scans and across days.

## Materials and Methods

### Data and Preprocessing

The minimally preprocessed and FIX de-noised rs-fMRI of the HCP S900 release (Van Essen et al., 2013) were downloaded. Before additional preprocessing, we calculated the mean, standard deviation, and temporal signal to noise ratio (tSNR) for each parcel from each individual. All 817 individuals with four complete rs-fMRI scans were preprocessed for a previous study (Yousefi et al., 2018). To summarize, each timeseries was demeaned and bandpass filtered (0.01–0.1 Hz) to maximize sensitivity to fluctuations related to neural activity and minimize contributions from noise. White matter, CSF and gray matter signals were regressed out. This is equivalent to global signal regression (GSR), which increased the similarity of rs-fMRI spatiotemporal structure across subjects in our prior work (Yousefi et al., 2018). The spatial dimension was reduced to 360 cortical parcels (Glasser et al., 2016) and each parcel’s timeseries was z-scored. Analysis was performed on the parcel level rather than at the vertex level to improve the signal-to-noise ratio further and to reduce computation times. As a complementary analysis, complexity metrics were also calculated for parcellated minimally-preprocessed data (no GSR; Supplementary Figure 5). All primary calculations were performed on the first scan from the first day (D1S1) for each subject. The second scan from the first day (D1S2) and first scan from the second day (D2S1) were used to examine reliability. Matrices were sorted so that parcels from the same networks (Yeo et al., 2011) were adjacent to each other using a simple rule that assigns each parcel to the network with which it has maximal overlap.

### Calculation of tSNR

Using the minimally-preprocessed data, the mean and standard deviation were calculated for the time course from each parcel. The tSNR was then given by the ratio of the mean to the standard deviation.

### Calculation of Power Spectra and Weighted Average Frequency

The power spectral density of each time course from each parcel for each subject was calculated using the Matlab pwelch function (8 segments, 50% overlap, Hamming window). Because the power spectrum is a vector, it is difficult to compare it directly to measures of complexity. We chose to characterize each power spectrum using a single value, the weighted average frequency (WAF):

WAF = $\frac{\sum p_{x}\,f_{x}}{\sum p_{x}}$ (1)

where P_*x*_ is the power in each frequency bin x and f_*x*_ is the frequency of the bin. The power spectra of interest tend to be dominated by low frequency components in the HCP data, and so the weighted average frequency tends to reflect the relative contribution of higher frequencies for each time course. Supplementary Figure 1 shows an example power spectral density plot and the resulting weighted average frequency. The weighted average frequency for the power spectrum was calculated for each parcel from each subject, resulting in a 817 × 360 matrix. For convenience of calculation, the weighted average frequency was set to the discrete value of the frequency at which the sum of weighted frequencies was greater than half of the sum over the whole distribution.

### Calculation of Complexity Metrics

Several metrics of complexity were calculated for each parcel from each subject. The first step of the calculation involves the estimation of a lower dimensional space that is “embedded” in the inherently high dimensional signal and captures its most prominent features. We used Matlab’s phase Space Reconstruction to estimate the dimensionality and the appropriate lag for a low dimensional representation of the signal. This algorithm uses the first minimum of average mutual information to estimate the embedding lag, and the false nearest neighbor algorithm to determine the embedding dimension. These values are used in the calculation of correlation dimension, approximate entropy, and Lyapunov exponent. The correlation Dimension function first generates a delay embedding of the signal, then is calculated as the slope of the correlation integral vs. the range of the radius of similarity. It provides an estimate of the chaotic complexity of the signal. The Lyapunov exponent measures the rate of divergence of trajectories in phase space and can be used as a measure of chaos in the system (lyapunovExponent). Approximate entropy measures regularity in a time series to quantify the level of complexity (approximateEntropy). For each metric, these calculation resulted in a 817 × 360 matrix.

### Variability Across Subjects/Parcels

To examine variability across subjects, the mean across all parcels was calculated for each individual, and the mean and standard deviation of the resulting distribution across individuals were recorded. To examine variability across parcels, the mean across all subjects was calculated for each parcel, and the mean and standard deviation of the resulting distribution across parcels were recorded.

### Variability Across Scans/Days

To examine consistency for both parcels and individuals, we first calculated the correlation coefficient between the full matrices from D1S1 and D1S2 (to examine short term consistency) and between the full matrices from D1S1 and D2S1 (to examine longer term consistency). We also assessed the reliability of the metrics between D1S1-D1S2 and D1S1-D2S1 using intraclass correlation (ICC). Specifically, we used a two-way mixed single score ICC(3,1) (Shrout and Fleiss, 1979), which is based on two-way ANOVA with the two scanning times as fixed effects and subjects as random effects and the unit of analysis is measurements obtained from each scanning time.

Because variations in basic signal properties are more prominent at the parcel level than at the individual level, we also calculated the correlation between the values averaged across individuals for each of the scans.

### Relationships Between Metrics

To determine whether complexity metrics (correlation dimension, Lyapunov exponent, and approximate entropy) provide additional information beyond the basic signal properties (mean, standard deviation, tSNR, and WAF), we first calculated the average across individuals to obtain the values for 360 parcels. Correlation between each basic property and each complexity metric was calculated and tested for significance. Bonferroni correction was applied to control for multiple comparisons (*n* = 12, requiring *p* < 0.0042 for significance with α = 0.05).

### Effects of Filtering and Noise on Metrics of Complexity

For comparison to the rs-fMRI data, we created 100 time series of 1200 points that were randomly sampled from a Gaussian distribution (randn in Matlab). These time series were bandpass filtered using three different passbands: 0.01–0.1 Hz, 0.1–0.2 Hz, and 0.01–0.2 Hz. We then calculated correlation dimension, approximate entropy, and Lyapunov exponent for each filtered time series to determine whether the average frequency or bandwidth of the signal affected the metrics of complexity. To examine the effects of noise, time series were bandpass filtered (0.01–0.1 Hz), then Gaussian noise was added with an amplitude of 0.2 or 0.5 times the standard deviation. Correlation dimension, approximate entropy, and Lyapunov exponent were calculated.

All code is available on the lab website^1^ and github^2^.

## Results

### Fundamental Signal Properties

**FIGURE 1:**
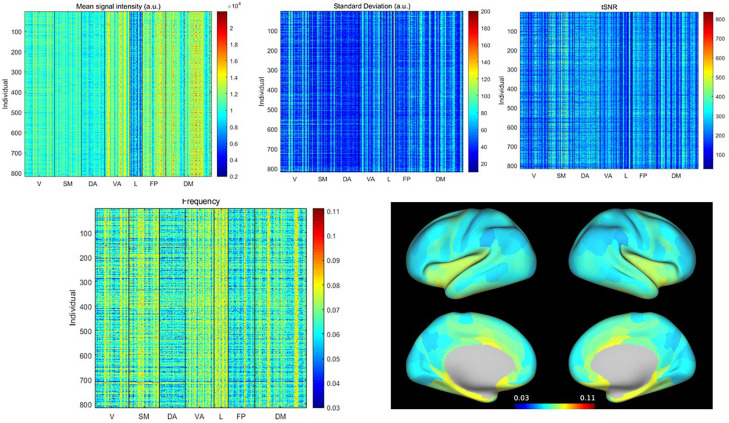
**(Top)** Mean, standard deviation and tSNR for all parcels from all individuals, sorted by network (V, visual; SM, somatomotor; DA, dorsal attention; VA, ventral attention; L, limbic; FP, frontoparietal; DM, default mode). **(Bottom)** Weighted average frequency from all parcels from all individuals, and weighted average frequency averaged across individuals, displayed on the brain surface. Variability is evident across both individuals and parcels. Limbic and ventral attention networks exhibit the highest weighted average frequencies, while the dorsal attention and frontoparietal networks exhibit the lowest. In other networks, there is a greater mix of areas with higher or lower weighted average frequencies.

The mean signal intensity, standard deviation, tSNR, and weighted average frequency for all subjects and all parcels are shown in Figure 1. Visually it is apparent that particular parcels tend to have higher or lower values across all individuals (vertical lines) and also that some individuals tend to have higher or lower values across all parcels (horizontal lines). Because the WAF is not commonly used to describe rs-fMRI signals, we compared it to standard metrics and obtained correlation values for the full parcel by individual matrices of 0.05 with the mean, 0.21 with standard deviation, and −0.15 with tSNR, indicating that the WAF provides complementary information to these metrics. The average WAF was 0.065 ± 0.01 Hz, well within the 0.01–0.08 Hz range widely used to define spontaneous BOLD fluctuations. Supplementary Figure 2 provides a histogram of the number of counts per frequency bin for each parcel across all scans, giving a visual demonstration of the distribution of the weighted average frequency. The limbic network exhibited low signal intensity, low tSNR and high WAF, while the dorsal attention network exhibited uniformly low WAF. Most other networks had a mixture of parcels with high and low values. To demonstrate the spatial distribution of these values, in Figure 1, WAF is displayed on a dilated brain (Van Essen et al., 2012).

To examine the source of the differences in WAF, the power spectra for the five parcels with the highest WAFs averaged across subjects [Piriform (Pir), Area 25, entorhinal and posterior orbitofrontal cortex (pOFC)] and the five parcels with the lowest WAFs averaged across subjects [prefrontal (PF) regions, Area 46] are shown in Supplementary Figure 3. It can be seen that the higher WAF reflects a power distribution that is smooth and broad, while the power for parcels with low WAF falls off more rapidly. Supplementary Figure 4 provides the power spectra for the five individuals with the highest and lowest WAFs. At the individual level, power spectra averaged across parcels for subjects with low WAF appear similar to the power spectra averaged across individuals for parcels with low WAF. For subjects with high WAF, however, the power spectra are less smooth and more likely to exhibit distinct peaks than for parcels with high WAF. Because motion has complex effects on rs-fMRI, we also examined the motion (as categorized as in Yousefi et al., 2018) for the individuals with the highest and lowest WAF. For the lowest WAFs, two individuals were in the high motion category [mean framewise displacement (FD) > 0.4 mm], two were in the moderate category (FD 0.2–0.4 mm), and one was in the group with the lowest motion (<0.12 mm FD). In contrast, for the five subjects with the highest WAFs, two were in the moderate category and three were in the lowest motion category. While this clearly does not rule out an effect of motion on frequency, it suggests that the frequency characteristics of an individual are not completely driven by motion.

### Complexity Metrics

**FIGURE 2:**
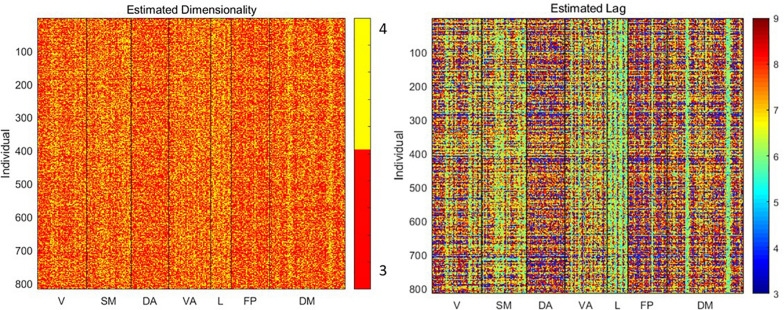
Estimated dimensionality and lag (in time points) for delay embedding of the signal from each parcel from each individual. Variability in lag especially can be observed across both individuals and parcels.

To understand the relationship between basic signal properties and measurements of complexity, we first estimated the optimal dimensionality and lag for delay embedding of the signal from each parcel from each subject. The resulting embedding is the basis for the other metrics of complexity examined here. As shown in Figure 2, the estimated dimensionality was either 3 or 4 for all scans. The lag time was more variable, ranging from 3 to 9 time points (∼2–6 s).

**FIGURE 3:**
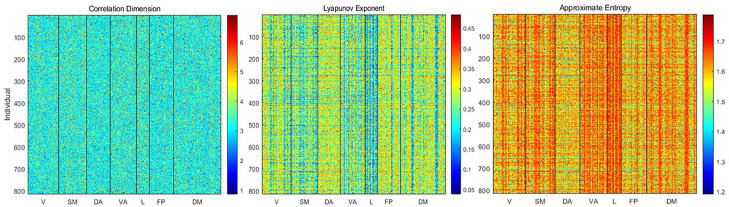
Correlation dimension, Lyapunov exponent, and approximate entropy for each parcel from each individual. Correlation dimension does not exhibit consistent variability across parcels or individuals, but the Lyapunov exponent and entropy show variability that is closely linked to the weighted average frequency. The Lyapunov exponent is anticorrelated with weighted average frequency, while entropy is correlated with weighted average frequency.

Using the delay embedding of each time course obtained with the estimated dimensionality and lag, we calculated the correlation dimension, Lyapunov exponent, and approximate entropy for each parcel from each individual (Figure 3). Correlation dimension has a mean of 3.26 ± 0.73 and showed relatively slight differences across either parcels or individuals. In contrast, the Lyapunov exponent (mean 0.27 ± 0.07) and approximate entropy (mean 1.6 ± 0.07) exhibited clear variability across both subjects and parcels.

### Variability Across Scans/Days

To examine the variability in basic metrics and complexity metrics, we compared the values from D1S1 to the values from D1S2 and D2S1 using Pearson correlation of the entire parcel by individual matrix and intraclass correlation [ICC(3,1)]. The results are summarized in Table 1. For all of the basic signal properties and most of the complexity metrics, the reliability is excellent, with ICC > 0.9. The exception is correlation dimension, which exhibits a lower ICC of 0.6–0.7.

**TABLE 1:** Correlation and intraclass correlation for the matrices of metrics calculated for every parcel from every individual using different rs-fMRI scans.

Metric	D1S1-D1S2	D1S1-D2S1
	CC/ICC	CC/ICC
Mean signal	0.94/0.98	0.86/0.95
Standard deviation	0.84/0.99	0.83/0.99
tSNR	0.82/0.996	0.80/0.995
Weighted average frequency	0.66/0.99	0.60/0.99
Correlation dimension	0.03/0.71	0.02/0.67
Approximate entropy	0.4/0.99	0.36/0.98
Lyapunov exponent	0.40/0.98	0.36/0.98

*D1, day 1; D2, day 2; S1, scan 1; S2, scan 2.*

We further examined the variability in the metrics across subjects and across parcels in different scans. The results are summarized in Tables 2, 3. All metrics for a given parcel appear to be highly reliable, while more variability is present at the individual level.

**TABLE 2:** Correlation between metrics calculated for every parcel, averaged across all individuals, using different rs-fMRI scans.

Metric	D1S1-D1S2	D1S1-D2S1
Mean signal	0.96	0.97
Standard deviation	0.92	0.93
tSNR	0.93	0.95
Weighted average frequency	0.98	0.99
Correlation dimension	0.91	0.91
Approximate entropy	0.98	0.99
Lyapunov exponent	0.97	0.99

**TABLE 3:** Correlation between metrics calculated for every individual, averaged across all parcels, using different rs-fMRI scans.

Metric	D1S1-D1S2	D1S1-D2S1
Mean signal	0.95	0.84
Standard deviation	0.79	0.70
tSNR	0.81	0.72
Weighted average frequency	0.77	0.54
Correlation dimension	0.55	0.55
Approximate entropy	0.71	0.52
Lyapunov exponent	0.75	0.54

### Relationships Between Metrics

**FIGURE 4:**
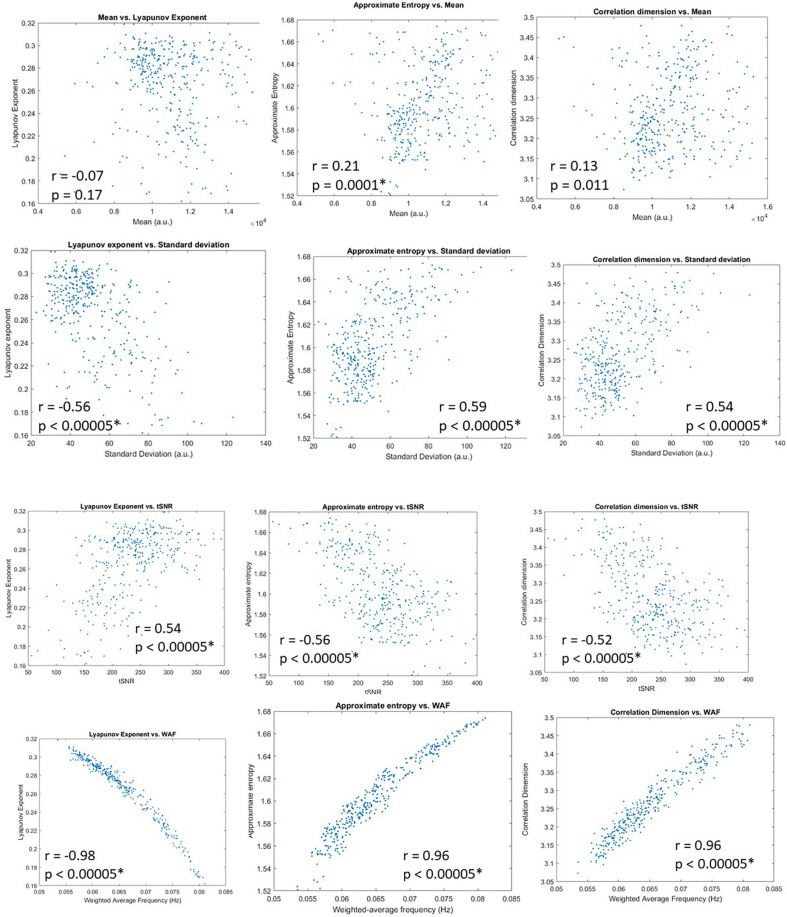
Scatterplots for the four basic signal properties (mean, standard deviation, tSNR, and weighted average frequency) vs. complexity metrics (Lyapunov exponent, approximate entropy, correlation dimension). All values were averaged across individuals. Correlation coefficients and *p*-values are given, with significance indicated by ^∗^. Weighted average frequency exhibits the closest relationship to complexity metrics.

Visual inspection of the parcel by individual matrices for the various metrics reveals commonalities and differences. To reduce the effect of noise and focus on parcel-wise relationships, we calculated correlation between each basic signal property and each complexity metric after averaging over individuals. Scatterplots are shown in Figure 4. The mean signal intensity was least correlated with the three metrics of complexity, and the only relationship that reached statistical significance was with entropy. Standard deviation and tSNR were significantly correlated with all metrics of complexity, with a correlation magnitude of ∼0.5. Given the relatively weak relationship between the mean signal intensity and complexity metrics, the relationship between metrics of complexity and tSNR is likely to be driven by their relationship to the standard deviation.

The correlation between the weighted average frequency and all complexity metrics was significant and surprisingly strong (-0.98 for Lyapunov exponent, 0.96 for approximate entropy and correlation dimension). Given the strong relationship observed parcelwise, we also calculated correlation between the full parcel by individual matrices. For WAF and Lyapunov exponent, the correlation was −0.64; for WAF and approximate entropy, the correlation was 0.65; for WAF and correlation dimension, the correlation was 0.17.

We also obtained complexity metrics directly from the minimally processed data (Supplementary Figure 5). Their values change substantially: −0.039 for Lyapunov exponent compared to 0.27 after additional preprocessing, 1.72 for approximate entropy compared to 1.6, and 3.6 for correlation coefficient compared to 3.26. However, the patterns of spatial variability were similar before and after additional preprocessing, suggesting that the underlying differences across parcels are preserved. For example, the limbic system continues to exhibit high entropy and low Lyapunov exponent in the minimally processed data.

### Effects of Filtering and Noise

For the randomly-generated and bandpass filtered time courses, neither the WAF nor the bandwidth affected correlation dimension, approximate entropy, or Lyapunov exponent (Table 4). However, the addition of noise had a marked effect, decreasing the Lyapunov exponent and increasing the approximate entropy (Table 5). The correlation dimension was relatively unaffected.

**TABLE 4:** Values of correlation dimension, Lyapunov exponent, and approximate entropy for randomly generated and bandpass filtered time courses.

Frequency range	Correlation dimension	Lyapunov exponent	Approximate entropy
0.01–0.1 Hz	1.95 ± 0.15	0.85 ± 0.26	1.73 ± 0.11
0.1–0.2 Hz	1.94 ± 0.14	0.84 ± 0.16	1.72 ± 0.10
0.01–0.2 Hz	1.96 ± 0.15	0.84 ± 0.21	1.72 ± 0.09
HCP data	3.26 ± 0.73	0.27 ± 0.07	1.60 ± 0.07

*Differences in average frequency and bandwidth have negligible impact on the complexity measures. Values for HCP data are shown for comparison.*

**TABLE 5:** Values of correlation dimension, Lyapunov exponent, and approximate entropy for randomly generated and bandpass filtered time courses with various levels of Gaussian noise added.

Added noise	Correlation dimension	Lyapunov exponent	Approximate entropy
None	1.97 ± 0.13	3.49 ± 0.06	0.62 ± 0.01
0.2σ	1.96 ± 0.15	1.32 ± 0.07	1.55 ± 0.04
0.5σ	1.95 ± 0.14	0.92 ± 0.06	1.7 ± 0.02
HCP data	3.26 ± 0.73	0.27 ± 0.07	1.60 ± 0.07

*Noise impacts the calculation of the Lyapunov exponent and approximate entropy, but has relatively little effect on the correlation dimension. Values for HCP data are shown for comparison.*

## Discussion

We showed that basic signal properties of the spontaneous BOLD fluctuations in the HCP data set vary across brain regions. Several metrics of signal complexity (correlation dimension, Lyapunov exponent, and approximate entropy) exhibit similar variability. While mean, standard deviation, and tSNR are significantly correlated to complexity metrics, the weighted average frequency exhibits the strongest relationship. However, analysis of filtered randomly-generated time courses shows that the frequency distribution alone is not sufficient to account for differences in complexity.

### Frequency Distribution of rs-fMRI Data

It has long been known that different frequency bands of the spontaneous BOLD fluctuations have differing contributions from noise and other processes. In an early paper, Cordes et al. (2001) showed that frequencies below 0.1 Hz accounted for most of the correlation between cortical areas. This concentration of functional information into the low frequency bands is also the basis for amplitude-based metrics like ALFF and fALFF (Zang et al., 2007; Zou et al., 2008). Note, however, that our analysis was performed on time series that were already bandpass filtered to 0.01–0.1 Hz, and so we are actually looking at the distribution of frequencies within the band typically used to measure functional connectivity.

### Specificity Within the Low Frequency Band

A number of other studies have shown that the frequency distribution within the low frequency band adds information about the functional organization of the brain. Using a wavelet-based analysis, Bajaj et al. (2013) showed that flow between nodes in higher frequencies is informative about activity at lower frequencies. Wang Y. et al., 2018 found that the right anterior insula exhibits a frequency-dependent relationship with large scale brain networks, and the network structure of the whole brain exhibits differences across frequencies (Thompson and Fransson, 2015). Similar to our study, Xue et al. (2014) found that higher frequencies were typically associated with lower functional connectivity, and that differences across frequencies were particularly prominent in limbic areas. We have previously used wavelet-based clustering to identify networks based on both their temporal and spectral characteristics, and there too the widely-recognized resting state networks were best defined at the lower end of the frequency spectrum (Billings et al., 2018). Moreover, clustering based on frequency information gave additional information to that available from traditional correlation analysis (Medda et al., 2016). Like our current work, these studies suggest that the power distribution of low frequency BOLD fluctuations holds information about the relative contributions of a variety of neural and non-neural processes.

A few research groups have begun to examine the relationship between measures of entropy and different rs-fMRI frequency bands. Using a modeling approach, Wang et al. found that multiscale entropy in the lower frequencies (0.02–0.087 Hz) in twenty subjects from the HCP data set was positively correlated with functional connectivity between those areas (Wang Y. et al., 2018). This is slightly discordant from our finding that higher WAFs are linked to higher entropy, but note that different measures of entropy were used and that the previous study did not look at the distribution of power within the low frequency band. In fact, they found that multiscale entropy at higher frequencies (0.347–0.694 Hz) was negatively related to functional connectivity, which could indicate that the high WAFs in our study may have been tied to higher power outside of the band used for functional connectivity analysis. In another study, Song et al. examined the correlation between sample entropy and fALFF, and found widespread anticorrelation between the two, especially in visual and somatomotor cortex. These prior reports of the frequency-dependence of complexity measures are in accordance with our current work.

### Complexity of rs-fMRI Signals

The brain can be described as a complex dynamical system. At the simplest level of analysis, researchers have examined the complexity of the time course of activity from a given region of interest, using measures like entropy. For methods that measure neural activity directly (e.g., EEG or local field potential recording), these types of analysis are expected to reflect the complexity of the brain’s activity and provide insight into the underlying dynamic structure of the brain. For rs-fMRI, however, the signal time course is only loosely tied to the underlying neural activity (Zhang et al., 2019a, b), partially because of the filtering imposed by neurovascular coupling and partially because of physiological and other noise. This raises the question of how well complexity measurements made on the rs-fMRI signal reflect the complexity of the underlying brain activity. Liu et al. (2019) recently addressed this question using voltage imaging in mice, which was then used to simulate rs-fMRI data. They found that some of the information captured in multi-scale entropy of the optical data was preserved in the simulated BOLD data. Extensive prior work has shown that despite the inherent filtering and noise, aspects of neural activity are preserved in the BOLD signal (Logothetis et al., 2001; Pan et al., 2011, 2013; Magri et al., 2012; Thompson et al., 2013, 2014), and it appears that this extends to complexity as well.

### Complexity vs. Frequency

There is no inherent reason that WAF should affect metrics of complexity. This is clear from the measurements made on the simulated data filtered into different frequency bands and from the moderate correlation between the full complexity and frequency matrices (∼0.6). We suspect that the relationship instead arises from common factors that influence both metrics. In this context, it is interesting that the highest correlations (> 0.9) are found between complexity measures and WAFs that are averaged across subjects to obtain values for each parcel. This suggests that there is something specific to each parcel, whether in the frequency of the spontaneous BOLD oscillations, the contribution of particular noise sources, or the overall level of SNR, that produces reliably distinct frequency distributions and measures of complexity. It seems likely that a combination of these contributions is responsible for the parcel-wise differences, rather than any single factor, and the weighting of the contributions may vary across brain areas.

Parcels with high WAFs are especially common in the limbic system and other areas near the base of the brain. These areas exhibit low signal intensity and low tSNR. In contrast, parcels near the top of the brain (e.g., the dorsal attention and frontoparietal networks) tend to have lower WAFs. Given the known variations in both image quality and coil sensitivity across the brain, the location of the parcels with high WAFs suggests that signal dropout, reduced coil sensitivity, and physiological noise may all be contributing factors. If this is the case, it would suggest that the relatively broader frequency profile in these regions indicates higher noise contributions that may obscure the desired signal. Our analysis of simulated data demonstrated that the addition of noise can substantially alter complexity metrics. The relatively low tSNR in the limbic system, for example, could account for the higher WAF and greater entropy (less predictability) observed there, along with reduced correlation. The lower Lyapunov exponent in the limbic system, which describes the tendency for divergence in trajectories that start from nearby points, may be the result of the manifold structure being degraded by noise. Effects like these that may result from fundamental aspects of image acquisition like coil sensitivity and signal dropout should be considered when interpreting any secondary signal features.

It is noteworthy that while WAF was very strongly correlated with complexity measures, tSNR was much more weakly related to complexity. This suggests that while complexity is affected by noise, it also reflects other processes that are captured by the WAF and which may be neuronal in origin. Further work with simultaneous measures of MRI and neural activity will be needed to better disentangle the relative contributions of noise and brain activity to metrics of complexity.

### Limitations

The WAF used as a metric in this study is a simplistic description of the actual frequency distribution. The primary difference in power distributions across parcels was in the width of the low frequency peak, suggesting that the full width half maximum of the distribution may also prove a useful metric.

There are arguments to be made for using unfiltered data for the calculation of correlation dimension, Lyapunov exponent, and approximate entropy, but for the primary analysis in this study, we have used data filtered to the range typically used for functional connectivity studies, with the rationale of minimizing noise. However, filtering undoubtedly discards relevant information in the higher frequencies along with the noise (Lee et al., 2013). It may prove possible to identify an “ideal” frequency profile from the full spectral range that could be used to identify and discard areas with high noise contributions, a topic worth further investigation.

Unsurprisingly, the full matrices of complexity metrics were less reliable across scans and across days than basic signal properties. The phase space reconstruction and subsequent calculation of entropy and Lyapunov exponent benefit from the use of large numbers of time points. A better estimate might be obtained by concatenating all four resting state scans, but at the expense of examining the reliability of the metrics.

## Conclusion

We have shown that the frequency distribution varies within the band of BOLD fluctuations used to map functional connectivity, particularly across parcels. Complexity metrics like Lyapunov exponent and approximate entropy show similar variation, suggesting that common factors impact both types of metrics. Given the location of the parcels with the highest WAFs, we believe that the differences partially reflect greater noise contributions. These fundamental sources of variation should be considered during the interpretation of measures of complexity.

## Data Availability Statement

The raw data supporting the conclusions of this article will be made available by the authors, without undue reservation, to any qualified researcher.

## Ethics Statement

Ethical review and approval was not required for the study on human participants in accordance with the local legislation and institutional requirements. Written informed consent for participation was not required for this study in accordance with the national legislation and the institutional requirements.

## Author Contributions

SK designed and executed the study. EM created the figures. BY performed the preprocessing. YG advised on statistical analysis. EM, XZ, W-JP, MN, NX, TL, and YG discussed the ideas and gave critical feedback on methods. All authors contributed to the article and approved the submitted version.

## Conflict of Interest

The authors declare that the research was conducted in the absence of any commercial or financial relationships that could be construed as a potential conflict of interest.

## Footnotes

1 https://sites.google.com/view/keilholz-lab/resources

2 https://github.com/keilholzMINDlab
