# Supplementary material for: Relationship Between Basic Properties of BOLD Fluctuations and Calculated Metrics of Complexity in the Human Connectome Project
Source: Front Neurosci. 2020 Sep 15;14:550923. doi: 10.3389/fnins.2020.550923 (PMC7522447; doi:10.3389/fnins.2020.550923)

Supplemental Material

Supplemental Figure 1. A power spectral density plot for one parcel from one subject. The red line indicates the original frequency distribution; the blue line indicates the weighted frequency distribution, and the black vertical line shows the weighted average frequency.

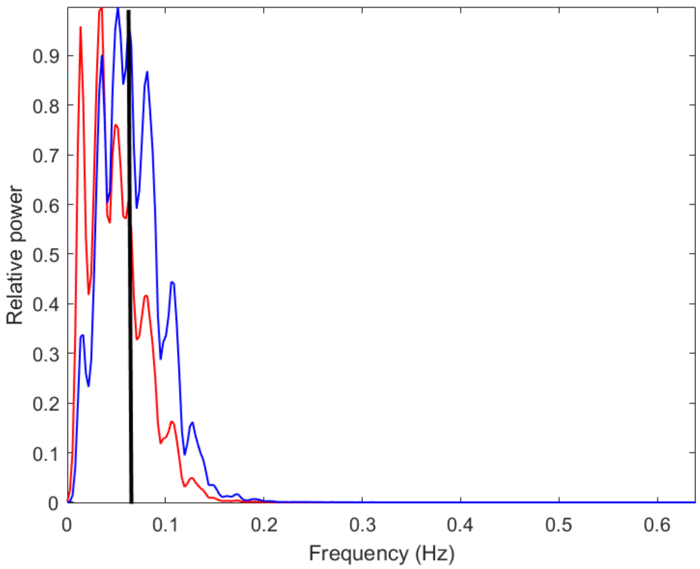

Supplemental Figure 2. (Left) Weighted average frequency for every parcel from every subject for Day 1 Scan 1. (Right) A histogram of the number of counts for each frequency bin for each parcel across all subjects and scans.

Day 1 Scan 1

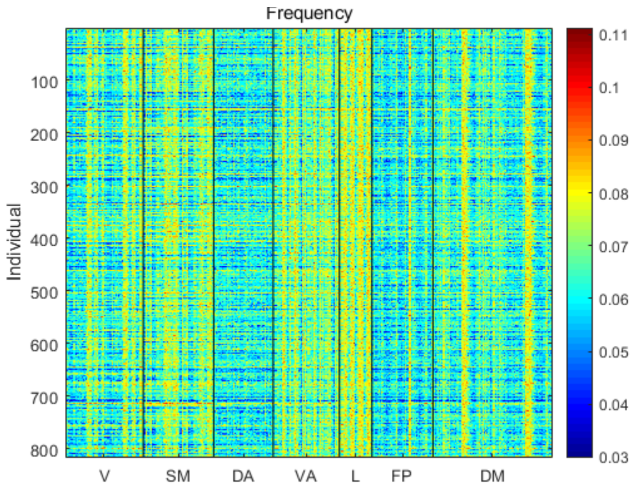

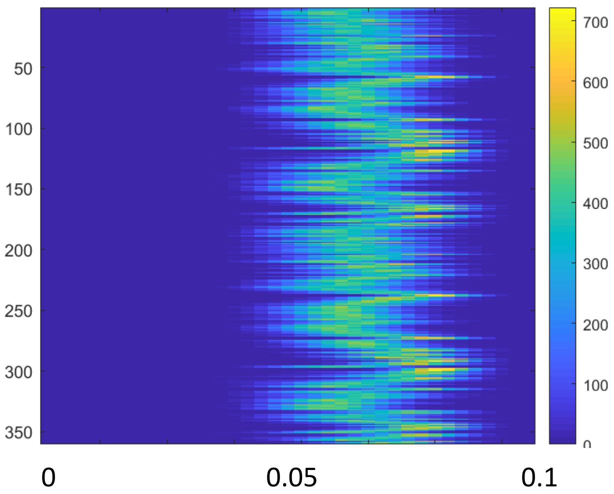

Histogram of frequency distribution for each parcel

Supplemental Figure 3a. Power spectra from the five parcels with the highest weighted average frequency (110, piriform cortex; 290, piriform cortex; 344, Area 25; 298, Entorhinal; 346, pOFC).

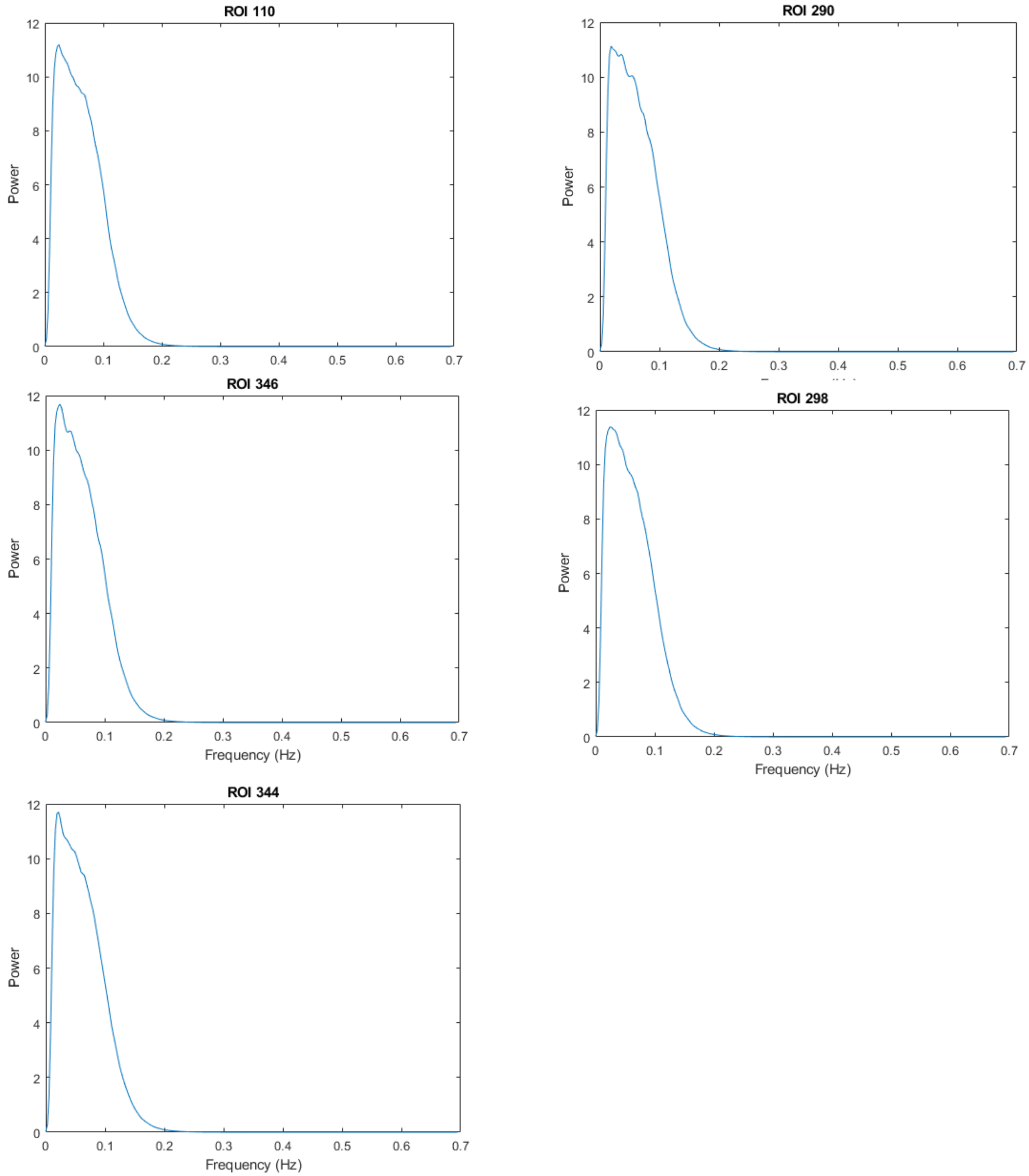

Supplemental Figure 3b. Power spectra for the five parcels with the lowest weighted average frequencies (148, Area PF; 328, PF; 84, Area 46; 264, Area 46; 149, PFm).

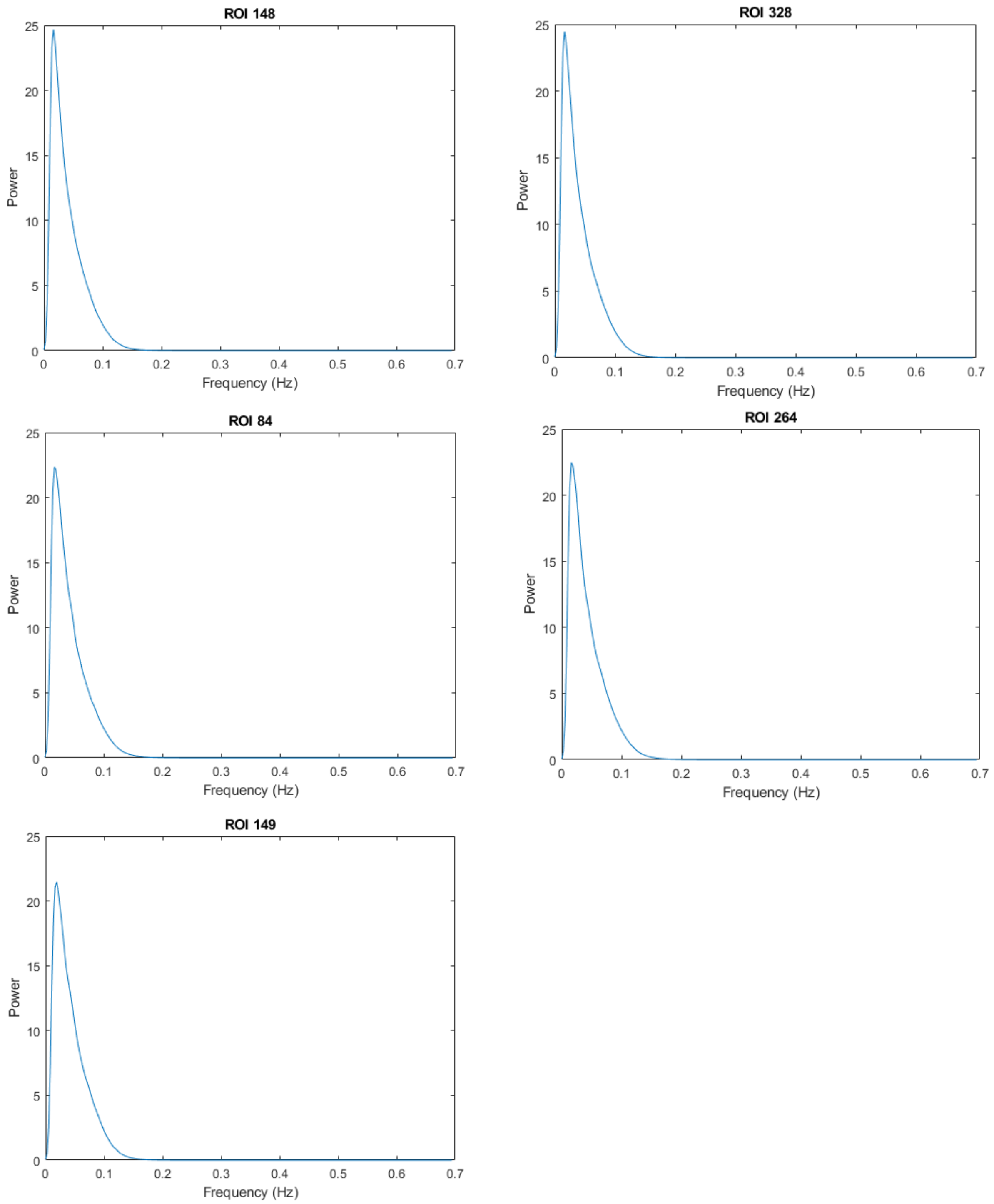

Supplemental Figure 4a. Power spectra from the five individuals with the highest weighted average frequencies.

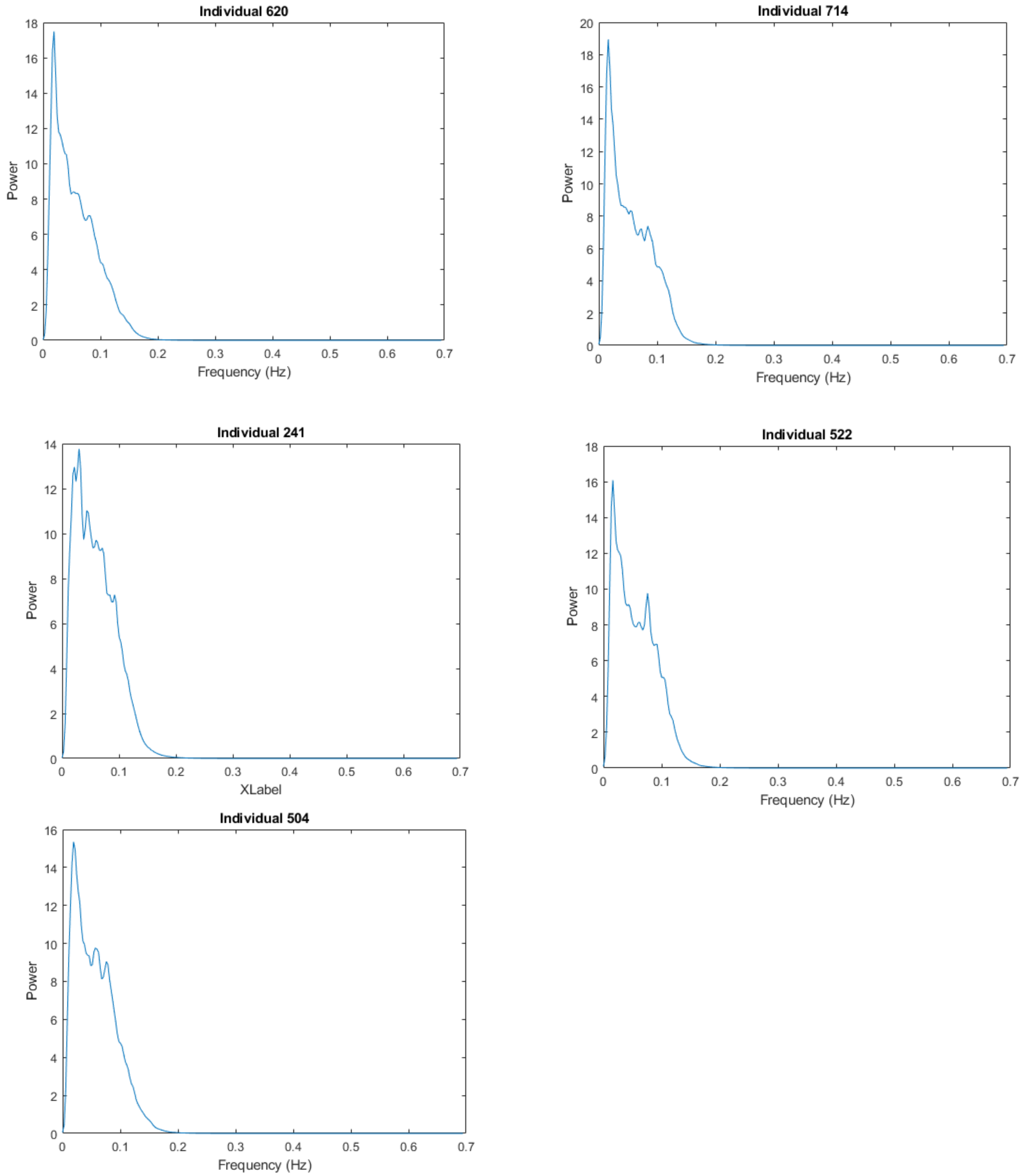

Supplemental Figure 4b. Power spectra from the five individuals with the lowest weighted average frequencies.

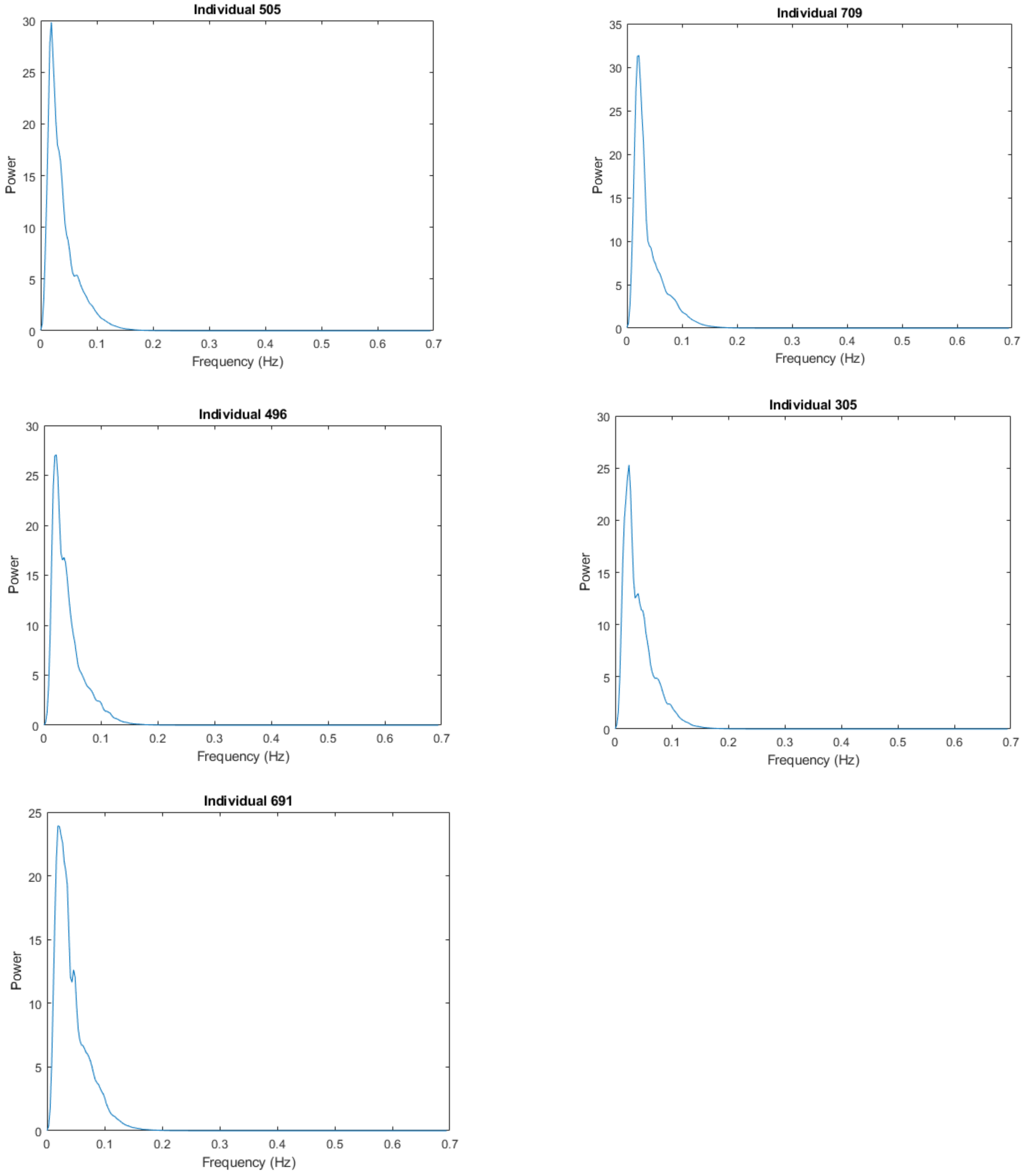

Supplemental Figure 5. In minimally pre-processed data (no global signal regression), complexity metrics have different values but parcel-level trends are preserved.

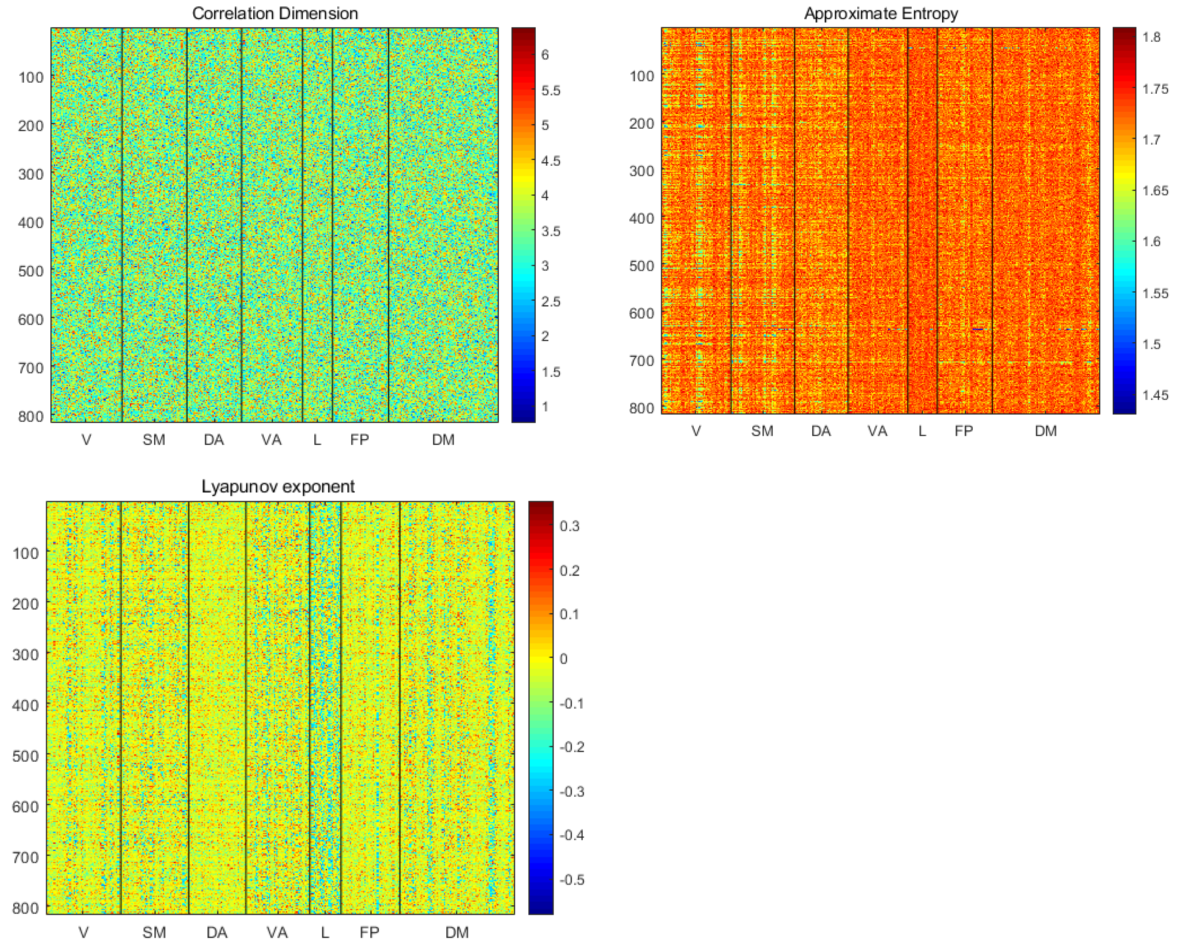

Supplement: Supplementary file 1 [file Image_1.pdf]
